# BioHackathon series in 2011 and 2012: penetration of ontology and linked data in life science domains

**DOI:** 10.1186/2041-1480-5-5

**Published:** 2014-02-05

**Authors:** Toshiaki Katayama, Mark D Wilkinson, Kiyoko F Aoki-Kinoshita, Shuichi Kawashima, Yasunori Yamamoto, Atsuko Yamaguchi, Shinobu Okamoto, Shin Kawano, Jin-Dong Kim, Yue Wang, Hongyan Wu, Yoshinobu Kano, Hiromasa Ono, Hidemasa Bono, Simon Kocbek, Jan Aerts, Yukie Akune, Erick Antezana, Kazuharu Arakawa, Bruno Aranda, Joachim Baran, Jerven Bolleman, Raoul JP Bonnal, Pier Luigi Buttigieg, Matthew P Campbell, Yi-an Chen, Hirokazu Chiba, Peter JA Cock, K Bretonnel Cohen, Alexandru Constantin, Geraint Duck, Michel Dumontier, Takatomo Fujisawa, Toyofumi Fujiwara, Naohisa Goto, Robert Hoehndorf, Yoshinobu Igarashi, Hidetoshi Itaya, Maori Ito, Wataru Iwasaki, Matúš Kalaš, Takeo Katoda, Taehong Kim, Anna Kokubu, Yusuke Komiyama, Masaaki Kotera, Camille Laibe, Hilmar Lapp, Thomas Lütteke, M Scott Marshall, Takaaki Mori, Hiroshi Mori, Mizuki Morita, Katsuhiko Murakami, Mitsuteru Nakao, Hisashi Narimatsu, Hiroyo Nishide, Yosuke Nishimura, Johan Nystrom-Persson, Soichi Ogishima, Yasunobu Okamura, Shujiro Okuda, Kazuki Oshita, Nicki H Packer, Pjotr Prins, Rene Ranzinger, Philippe Rocca-Serra, Susanna Sansone, Hiromichi Sawaki, Sung-Ho Shin, Andrea Splendiani, Francesco Strozzi, Shu Tadaka, Philip Toukach, Ikuo Uchiyama, Masahito Umezaki, Rutger Vos, Patricia L Whetzel, Issaku Yamada, Chisato Yamasaki, Riu Yamashita, William S York, Christian M Zmasek, Shoko Kawamoto, Toshihisa Takagi

**Affiliations:** 1 Database Center for Life Science, Research Organization of Information and Systems, 2-11-16, Yayoi, Bunkyo-ku, Tokyo 113-0032, Japan; 2 Centro de Biotecnología y Genómica de Plantas UPM-INIA (CBGP), Universidad Politécnica de Madrid, Campus Montegancedo, 28223-Pozuelo de, Alarcón, Spain; 3 Department of Bioinformatics, Faculty of Engineering, Soka University, 1-236 Tangi-machi, Hachioji, Tokyo 192-8577, Japan; 4 National Institute of Informatics, JST PRESTO, 2-1-2 Hitotsubashi, Chiyoda-ku, Tokyo 101-8430, Japan; 5 Department of Electrical Engineering (ESAT/SCD), University of Leuven, Kasteelpark Arenberg 10, Leuven 3001, Belgium; 6 iMinds Future Health Department, University of Leuven, Kasteelpark Arenberg 10, Leuven 3001, Belgium; 7 Department of Biology, Norwegian University of Science and Technology (NTNU), Høgskoleringen 5, Trondheim N-7491, Norway; 8 Institute for Advanced Biosciences, Keio University, Endo 5322, Fujisawa, Kanagawa 252-0882, Japan; 9 Silicon Cat Ltd. 5 York Road, London HA6 1JJ, UK; 10 Ontario Institute for Cancer Research, 101 College Street, Suite 800, Toronto, Ontario M5G 0A3, Canada; 11 SIB Swiss Institute of Bioinformatics, CMU, rue Michel Servet, Geneve 4 1211, Switzerland; 12 Integrative Biology Program, Istituto Nazionale Genetica Molecolare, Milan 20122, Italy; 13 The Alfred Wegener Institute, Helmholtz Centre for Polar and Marine Research, Am Handelshafen 12, Bremerhaven 27570, Germany; 14 Biomolecular Frontiers Research Centre, Macquarie University, North Ryde NSW 2109, Australia; 15 National Institute of Biomedical Innovation, 7-6-8 Asagi Saito, Ibaraki-City, Osaka 567-0085, Japan; 16 National Institute for Basic Biology, National Institutes of Natural Sciences, Nishigonaka 38, Myodaiji, Okazaki, Aichi 444-8585, Japan; 17 The James Hutton Institute, Invergowrie, Dundee DD2 5DA, UK; 18 Center for Computational Pharmacology, University of Colorado Denver School of Medicine, Aurora, CO 80045, USA; 19 School of Computer Science, The University of Manchester, Oxford Road M13 9PL, UK; 20 Department of Biology, Institute of Biochemistry, School of Computer Science, Carleton University, 1125 Colonel By Drive, Ottawa, Ontario K1S 5B6, Canada; 21 Center for Information Biology, National Institute of Genetics, Research Organization of Information and Systems, 1111 Yata, Mishima, Shizuoka 411-08540, Japan; 22 INTEC Inc, 1-3-3 Shinsuna, Koto-ku, Tokyo 136-8637, Japan; 23 Genome Information Research Center, Research Institute for Microbial Diseases, Osaka University, 3-1 Yamadaoka, Suita, Osaka 565-0871, Japan; 24 Department of Physiology, Development and Neuroscience, University of Cambridge, Downing Street, Cambridge CB2 3EG, UK; 25 Atmosphere and Ocean Research Institute, the University of Tokyo, 5-1-5 Kashiwanoha, Kashiwa, Chiba 277-8564, Japan; 26 Computational Biology Unit, Uni Computing and Department of Informatics, University of Bergen, Thormøhlensgate 55, Bergen 5008, Norway; 27 Korea Institute of Science Technology and Information, 245 Daehangno, Yuseong, Daejeon 305-806, Korea; 28 Department of Biotechnology, Bioinformation Engineering Laboratory, Graduate School of Agricultural and Life Sciences, The University of Tokyo, 1-1-1, Yayoi, Bunkyo-ku, Tokyo 113-8657, Japan; 29 Bioinformatics Center, Institute for Chemical Research, Kyoto University, Gokasho, Uji, Kyoto 611-0011, Japan; 30 EMBL-European Bioinformatics Institute, Wellcome Trust Genome Campus, Hinxton, Cambridge CB10 1SD, UK; 31 National Evolutionary Synthesis Center (NESCent), 2024 W. Main St, Durham, NC, USA; 32 Justus-Liebig-University Giessen, Institute of Veterinary Physiology and Biochemistry, Frankfurter Str. 100, Giessen 35392, Germany; 33 MAASTRO Clinic, Maastricht, Postbus 3035, Maastricht 6202 NA, The Netherlands; 34 Department of Biological Information, Graduate School of Bioscience and Biotechnology, Tokyo Institute of Technology, 4259 B-36, Nagatsuta-cho, Midori-ku, Yokohama 226-8501, Japan; 35 Center for Knowledge Structuring, The University of Tokyo, 7-3-1 Hongo, Bunkyo-ku, Tokyo, Japan; 36 Biomedicinal Information Research Center, National Institute of Advanced Industrial Science and Technology, Aomi 2-4-7, Koto-ku, Tokyo 135-0064, Japan; 37 Next Generation Systems Core Function Unit, Eisai Product Creation Systems, Eisai Co., Ltd, 5-3-1 Toukoudai, Tsukuba, Ibaraki 300-2635, Japan; 38 Research Center for Medical Glycoscience, National Institute of Advanced Industrial Science and Technology (AIST) 1-1-1 Umezono, Tsukuba, Ibaraki 305-8568, Japan; 39 Department of Bioclinical informatics, Tohoku Medical Megabank Organization, Tohoku University, Seiryo-cho 4-1, Aoba-ku, Sendai-shi, Miyagi 980-8575, Japan; 40 Graduate School of Information Sciences (GSIS), Tohoku University, 6-3-09 Aoba, Aramaki-aza, Aoba-ku, Sendai, Miyagi, 980-8575, Japan; 41 Niigata University Graduate School of Medical and Dental Sciences, 1-757 Asahimachi-dori, Chuo-ku, Niigata 951-8510, Japan; 42 Biomolecular Frontiers Research Centre, Macquarie University, North Ryde, NSW 2109, Australia; 43 Laboratory of Nematology, Droevendaalsesteeg 1, Wageningen University, Wageningen, Netherlands; 44 Department of Biochemistry and Molecular Biology, The University of Georgia, 315 Riverbend Road, Athens, GA 30602, USA; 45 Oxford e-Research Center, University of Oxford, Oxford OX1 3QG, UK; 46 Digital Enterprise Research Institute, IDA Business Park, Lower Dangan, Galway, Ireland; 47 intelliLeaf.com, Cambridge, UK; 48 CeRSA, Parco Tecnologico Padano, Lodi 26900, Italy; 49 Zelinsky Institute of Organic Chemistry, Russian Academy of Sciences, Leninsky prospekt 47, Moscow 119991, Russia; 50 Division of International Cooperative Research, Research Center for Ethnomedicine, Institute of Natural Medicine, University of Toyama, 2630 Sugitani, Toyama 930-0194, Japan; 51 Naturalis Biodiversity Center, Postbus 9517, Leiden 2300 RA, the Netherlands; 52 Stanford Center for Biomedical Informatics Research, Stanford University, Stanford, CA 94305-5479, USA; 53 Laboratory of Glyco-organic Chemistry, The Noguchi Institute, 1-8-1 Kaga, Itabashi-ku, Tokyo 173-0003, Japan; 54 Tohoku Medical Megabank Organization, Tohoku University, Research Building No.3, 6-3-09 Aoba, Aramaki-aza, Aoba-ku, Sendai, Miyagi 980-8575, Japan; 55 Program on Bioinformatics and Systems Biology, Sanford-Burnham Medical Research Institute, La Jolla, CA 92037, USA; 56 Department of Computational Biology, University of Tokyo, Kashiwa, Chiba 277-8568, Japan

**Keywords:** BioHackathon, Bioinformatics, Semantic Web, Web services, Ontology, Visualization, Knowledge representation, Databases, Semantic interoperability, Data models, Data sharing, Data integration

## Abstract

The application of semantic technologies to the integration of biological data and the interoperability of bioinformatics analysis and visualization tools has been the common theme of a series of annual BioHackathons hosted in Japan for the past five years. Here we provide a review of the activities and outcomes from the BioHackathons held in 2011 in Kyoto and 2012 in Toyama. In order to efficiently implement semantic technologies in the life sciences, participants formed various sub-groups and worked on the following topics: Resource Description Framework (RDF) models for specific domains, text mining of the literature, ontology development, essential metadata for biological databases, platforms to enable efficient Semantic Web technology development and interoperability, and the development of applications for Semantic Web data. In this review, we briefly introduce the themes covered by these sub-groups. The observations made, conclusions drawn, and software development projects that emerged from these activities are discussed.

## Introduction

In life sciences, the Semantic Web is an enabling technology which could significantly improve the quality and effectiveness of the integration of heterogeneous biomedical resources. The first wave of life science Semantic Web publishing focused on availability - exposing data as RDF without significant consideration for the quality of the data or the adequacy or accuracy of the RDF model used. This allowed a proliferation of proof-of-concept projects that highlighted the potential of Semantic technologies. However, now that we are entering a phase of adoption of Semantic Web technologies in research, quality of data publication must become a serious consideration. This is a prerequisite for the development of translational research and for achieving ambitious goals such as personalized medicine.

While Semantic technologies, in and of themselves, do not fully solve the interoperability and integration problem, they provide a framework within which interoperability is dramatically facilitated by requiring fewer pre-coordinated agreements between participants and enabling unanticipated *post hoc* integration of their resources. Nevertheless, certain choices must be made, in a harmonized manner, to maximize interoperability. The yearly BioHackathon series [1-3] of events attempts to provide the environment within which these choices can be explored, evaluated, and then implemented on a collaborative and community-guided basis. These BioHackathons were hosted by the National Bioscience Database Center (NBDC) [4] and the Database Center for Life Science (DBCLS) [5] as a part of the Integrated Database Project to integrate life science databases in Japan. In order to take advantage of the latest technologies for the integration of heterogeneous life science data, researchers and developers from around the world were invited to these hackathons.

This paper contains an overview of the activities and outcomes of two highly interrelated BioHackathon events which took place in 2011 [6] and 2012 [7]. The themes of these two events focused on representation, publication, and exploration of bioinformatics data and tools using standards and guidelines set out by the Linked Data and Semantic Web initiatives.

## Review

**Figure 1:**
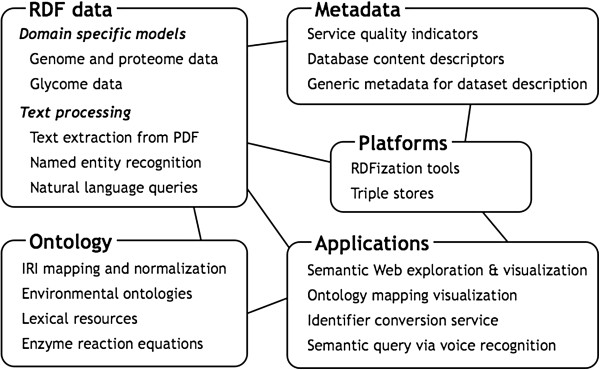
**Overview of categories and topics raised during the BioHackathons of 2011 and 2012.** Lines between the boxes represent semantic relationships between categories.

Semantic Web technologies are formalized as World Wide Web consortium (W3C) standards aimed at creating general-purpose, long-lived data representation, exchange, and integration formats that replace current *ad hoc* solutions. However, because they are general-purpose standards, many issues need to be addressed and agreed-upon by the community in order to apply them successfully to the integration and interoperability problems of the life science domain. Therefore, participants of the BioHackathons fall into sub-groups of interest within the life sciences, representing the specific needs and strengths of their individual communities within the broader context of life science informatics. Though there were multiple specific activity groups under each of the following headings, and there was overlap and cross-talk between the activities of each group, we will organize this review under the five general categories of: RDF data, Ontology, Metadata, Platforms and Applications (Figure 1). Results and issues raised by each group are briefly summarized in the Table 1. We also note that many groups have or will publish their respective outcomes in individual publications.

**Table 1:** Summary of investigated issues and results covered during BioHackathons 2011 and 2012

	
**RDF data**	
	***Domain specific models***
	*Genome and proteome data*
	***Issue***: No standard RDF data model and tools existed for major genomic data
	***Result***: Created FALDO, INSDC, GFF, GVF ontologies and developed converters
	***Software***: Converters are now packaged in the BioInterchange tool; improved PSICQUIC service
	*Glycome data*
	***Issue***: Glycome and proteome databases are not effectively linked
	***Result***: Developed a standard RDF representation for carbohydrate structures by BCSDB, GlycomeDB, GLYCOSCIENCES.de, JCGGDB, MonosaccharideDB, RINGS, UniCarbKB and UniProt developers
	***Software***: RDFized data from these databases, stored them in Virtuoso and tested SPARQL queries among the different data resources
	***Text processing***
	*Text extraction from PDF and metadata retrieval*
	***Issue***: Text for mining is often buried in the PDF formatted literature and requires preprocessing
	***Result***: Incorporated a tool for text extraction combined with a metadata retrieval service for DOIs or PMIDs
	***Software***: Used PDFX for text extraction; retrieved metadata by the TogoDoc service
	*Named entity recognition and RDF generation*
	***Issue***: No standard existed for combining the results of various NER tools
	***Result***: Developed a system for combining, viewing, and editing the extracted gene names to provide RDF data
	***Software***: Extended SIO ontology for NER and newly developed the BioInterchange tool for RDF generation
	*Natural language query conversion to SPARQL*
	***Issue***: Automatic conversion of natural language queries to SPARQL queries is necessary to develop a human friendly interface
	***Result***: Incorporated the SNOMED-CT dataset to answer biomedical questions and improved linguistic analysis
	***Software***: Improved the in-house LODQA system; used ontologies from BioPortal
**Ontology**	
	***IRI mapping and normalization***
	***Issue***: IRIs for entities automatically generated by BioPortal do not always match with submitted RDF-based ontologies
	***Result***: Normalized IRIs in the BioPortal SPARQL endpoint as either the provider IRI, the Identifiers.org IRI, or the Bio2RDF IRI
	***Software***: Used services of BioPortal, the MIRIAM registry, Identifires.org and Bio2RDF
	***Environmental ontologies for metagenomics***
	***Issue***: Semantically controlled description of a sample’s original environment is needed in the domain of metagenomics
	***Result***: Developed the Metagenome Environment Ontology (MEO) for the MicrobeDB project
	***Software***: References the Environment Ontology (EnvO) and other ontologies
	***Lexical resources***
	***Issue***: Standard machine-readable English-Japanese / Japanese-English dictionaries are required for multilingual utilization of RDF data
	***Result***: Developed ontology for LSD to serialize the lexical resource in RDF and published it at a SPARQL endpoint
	***Software***: Data provided by the Life Science Dictionary (LSD) project
	***Enzyme reaction equations***
	***Issue***: New ontology must be developed to represent incomplete enzyme reactions which are not supported by IUBMB
	***Result***: Designed semantic representation of incomplete reactions with terms to describe chemical transformation patterns
	***Software***: Obtained data from the KEGG database and the result is available at GenomeNet
**Metadata**	
	***Service quality indicators***
	***Issue***: Quality of the published datasets (SPARQL endpoints) is not clearly measured
	***Result***: Measured the availability, response time, content amount and other quality metrics of SPARQL endpoints
	***Software***: Web site is under development to illustrate the summary of periodical measurements
	***Database content descriptors***
	***Issue***: Uniform description of the core attributes of biological databases should be semantically described
	***Result***: Developed the RDF Schema for the BioDBCore and improved the BioDBCore Web interface for submission and retrieval
	***Software***: Evaluated identifiers for DBs in NAR, DBpedia, Identifiers.org and ORCID and vocabularies from Biositemaps, EDAM, BRO and OBI
	***Generic metadata for dataset description***
	***Issue***: Database catalogue metadata needs to be machine-readable for enabling automatic discovery
	***Result***: Conventions to describe the nature and availability of datasets will be formalized as a community agreement
	***Software***: Members from the W3C HCLS, DBCLS, MEDALS, BioDBCore, Biological Linked Open Data, Biositemaps, Uniprot, Bio2RDF, Biogateway, Open PHACTS, EURECA, and Identifiers.org continue the discussion in teleconferences
**Platforms**	
	***RDFization tools***
	***Issue***: RDF generation tools supporting various data formats and data sources are not yet sufficient
	***Result***: Tools to generate RDF from CSV, TSV, XML, GFF3, GVF and other formats including text mining results were developed
	***Software***: BioInterchange can be used as a tool, Web services and libraries; bio-table is a generic tool for tabular data
	***Triple stores***
	***Issue***: Survey is needed to test scalability of distributed/cluster-based triple stores for multi-resource integration
	***Result***: Hadoop-based and Cluster-based triple stores were still immature and federated queries on OWLIM-SE was still inefficient
	***Software***: HadoopRDF, SHARD and WebPIE for Hadoop-based triple stores; 4store and bigdata for Cluster-based triple stores
**Applications**	
	***Semantic Web exploration and visualization***
	***Issue***: Interactive exploration and visualization tools for Semantic Web resources are required to make effective queries
	***Result***: Tools are reviewed from viewpoints of requirements and availability, features, assistance and support, technical aspects, and specificity to life sciences use cases
	***Software***: More than 30 tools currently available are reviewed and classified for benchmarking and evaluations in the future
	***Ontology mapping visualization***
	***Issue***: Visualization of ontology mapping is required to understand how different ontologies with relating concepts are interconnected
	***Result***: Ontology mappings of all BioPortal ontologies and a subset of BioPortal ontologies suitable for OntoFinder/Factory were visualized
	***Software***: Applicability of Google Fusion Tables and Gephi were investigated
	***Identifier conversion service***
	***Issue***: Multiple synonyms for the same data inhibits cross-resource querying and data mining
	***Result***: Developed a new service to extract cross references from UniProt and KEGG databases, eliminate redundancy and visualize the result
	***Software***: G-Links resolves and retrieves all corresponding resource URIs
	***Semantic query via voice recognition***
	***Issue***: Intuitive search interface similar to “Siri for biologists” would be useful
	***Result***: Developed a context-aware virtual research assistant Genie which recognizes spoken English and replies in a synthesized voice
	***Software***: The G-language GAE, G-language Maps, KBWS EMBASSY and EMBOSS, and G-Links are used for Genie

### RDF data

In terms of RDF data generation, data were generated for genomic and glycomic databases (domain-specific models) and from the literature using text processing technologies. We describe these two subcategories here.

#### *Domain specific models*

##### 

**Genome and proteome data** Due to the high-throughput generation of genomic data, it is of high priority to generate RDF models for both nucleotide sequence annotations and amino acid sequence annotations. Up to now, nucleotide sequence annotations are provided in a variety of formats such as the International Nucleotide Sequence Database Collaboration (INSDC) [8], Generic Feature Format (GFF) [9] and Genome Variation Format (GVF) [10]. By RDFizing this information, all of the annotations from various sequencing projects can be integrated in a straightforward manner. This would in turn accommodate the data integration requirements of the H-InvDB [11]. In general, due to the large variety of genomic annotations possible, it was decided that in the first iteration of a genomic RDF model, opaque Universally Unique IDentifiers (UUIDs) are to be used to represent sequence features. Each UUID would then be typed with its appropriate ontology, such as Sequence Ontology (SO), and sequence location would be specified using Feature Annotation Location Description Ontology (FALDO) [12,13]. FALDO was newly developed at the BioHackathon 2012 by representatives of UniProt [14], DDBJ [15] and genome scientists for the purpose of generically locating regions on the biological sequences (e.g., modification sites on a protein sequence, fuzzy promoter locations on a DNA sequence etc.). A locally-defined vocabulary was used to annotate other aspects such as sequence version and synonymy. Thus, a generic system for nucleotide and amino acid sequence annotations could be proposed. Converters were also developed that would output compatible RDF documents, such as HMMER3 [16], GenBank/DDBJ [17], GTF [18] and GFF2OWL [19]. The RDF output for Proteomics Standard Initiative Common QUery InterfaCe (PSICQUIC) [20], a tool to retrieve molecular interaction data from multiple repositories with more than 150 milion interactions available at the time of writing, was modified during the Biohackathon 2011 to improve the mapping of identifiers and ontologies. Identifiers.org was chosen as the provider for the new IRIs for the interacting proteins and ontology terms to allow a better integration with other sources. PSICQUIC RDF output is based on the popular BioPAX format [21] for interactions and pathways.

##### 

**Glycome data** The Glycomics working group consisted of developers from the major glycomics databases including Bacterial Carbohydrate Structure Database (BCSDB) [22], GlycomeDB [23,24], GLYCOSCIENCES.de [25], Japan Consortium for Glycobiology and Glycotechnology Database (JCGGDB) [26], MonosaccharideDB [27], Resource for INformatics of Glycomes at Soka (RINGS) [28], and UniCarbKB [29]. These databases contain information about glycan structures, or complex carbohydrates, which are often covalently linked to proteins forming glycoproteins. The connections between glycomics and proteomics databases are required to accurately describe the properties and potential biological functions of glycoproteins. In order to establish such a connection this working group cooperated with UniProt developers present at the BioHackathon to agree upon and develop a standard RDF representation for carbohydrate structures, along with the relevant biological and bibliographic annotations and experimental evidence. Data from the individual databases have been exported in the newly developed RDF format (version 0.1) and stored in a triple store, allowing for cross-database queries. Several proof-of-concept queries were tested to show that federated queries could be made across multiple databases to demonstrate the potential for this technology in glycomics research. For example, both UniProt and JCGGDB are important databases in their respective domains of protein sequences and glycomics data. Moreover, UniCarbKB is becoming an important glycomics resource as well. However, since UniCarbKB is not linked with JCGGDB, a SPARQL query was described to find the JCGGDB entries for each respective UniCarbKB entry. Aoki-Kinoshita et al., 2013 [30] this was made possible by the integration of UniCarbKB, JCGGDB and GlycomeDB data, which served as the link between the former two datasets. This would not have been possible without agreement upon the standardization of the pertinent glycomics data in each database, discussed at BioHackathons.

#### *Text processing*

The Data Mining and Natural Language Processing (NLP) groups focused their efforts in two primary domains: information extraction from scientific text - particularly from PDF articles - in the form of ontology-grounded triples, and the conversion of natural language questions into triples and/or SPARQL queries. Both of these were pursued with an eye to standardization and interoperability between life science databases.

##### 

**Text extraction from PDF and metadata retrieval** The first step in information extraction is ensuring that accurate plain-text representations of scientific documents are available. A widely recognized “choke point” that inhibits the processing and mining of vast biomedical document stores has been the fact that the bulk of information within them is often available only as PDF-formatted documents. Access to this information is crucial for a variety of needs, including accessibility to model organism database curators and the population of RDF triple stores. In confronting this issue, the BioHackers worked on a novel software project called PDFX [31,32], which automatically converts the PDF scientific articles to XML form. The general use case was to include PDFX as a pre-processing step within a wide variety of more involved processing pipelines, such as the additional concerns of the BioHackathon data mining and NLP groups presented next. Complementing text extraction from PDF documents, when this process is employed, it also becomes necessary to retrieve relevant metadata information. This was done using DBCLS’s TogoDoc [33] literature management and recommendation system, which detects the Digital Object Identifier (DOI) or PubMed identifiers of PDF submissions in order to retrieve metadata information such as MeSH terms and make recommendations to users.

##### 

**Named entity recognition and RDF generation** Once text is in processable form, the next phase of information extraction is entity recognition within the text. The field of gene name extraction suffers from a prevalence of diverse annotation schemata, ontologies, definitions of semantic classes, and standards regarding where the edges of gene names should be marked within a corpus (an annotated collection of topic-specific text). In 2011, the NLP/text mining group worked on an application for combining, viewing and editing the outputs of a variety of gene-mention-detection systems, with the goal of providing RDF outputs of protein/gene annotation tools such as GNAT [34], GeneTUKit [35], and BANNER [36]. The Annotation Ontology was used to represent these metadata. However, at the 2012 event, the SIO ontology [37] was extended to enable representation of entity-recognition outputs directly in RDF: resources were described in terms of a number of novel relation types (properties) and incorporated in an inheritance and partonymy hierarchy. Using these various components as a proof of concept, the NLP sub-group began developing a generic RDFization framework, BioInterchange [38], comprised of three pipelined steps - data deserialization, object model generation, and RDF serialization - to enable easy data conversion into RDF with automatic ontological mappings primarily to SIO and secondarily to other ontologies.

##### 

**Natural language query conversion to SPARQL** The final activity within the NLP theme was the conversion of natural language queries to SPARQL queries. SPARQL queries are a natural interface to RDF triple-store endpoints, but they remain challenging to construct, even for those with intimate knowledge of the target data schema. It would be easier, for example, to enable users to ask a question such as “What is the sequence length for human TP53?” and receive an answer from the UniProt database, based on a SPARQL query that the system constructs automatically. A pre-existing tool from the DBCLS that can accomplish natural-language-to-SPARQL conversion was targeted and customized for the SNOMED-CT [39] dataset in BioPortal [40]. A large set of natural language test queries were developed, and for a subset of those queries the post-conversion output was analyzed and compared to a manually created gold standard output; subsequently, the group undertook a linguistic analysis of what conversions would have to be carried out in order to transform the current system output to the gold standard. These efforts included using natural language generation technology to build a Python solution that generates hundreds of morphological and syntactic variants of various natural language question types.

### Ontology

#### *IRI mapping and normalization*

The first step in any semantic integration activity is to agree on the identifiers for various concepts. BioPortal, a central repository for biomedical ontologies, allows users to download original ontology files in a variety of formats (OWL [41], OBO [42], etc.), but also makes these ontologies available using RDF through a Web service and SPARQL endpoint [43]. In RDF, entities (classes, relations and individuals) are identified using an Internationalized Resource Identifier (IRI); however, the identifiers that are automatically generated by BioPortal do not always match with those used in submitted RDF-based ontologies, thereby impeding integration across ontologies. Moreover, since ontologies are also used to semantically annotate biomedical data, there is a lack of semantic integration between data and ontology. BioHackathon activities included surveying, mapping, and normalizing the IRIs present in the RDF-based ontologies found in the BioPortal SPARQL endpoint to a canonical set of IRIs in a custom dataset and namespace registry, primarily used by the Bio2RDF project [44]. This registry is being integrated with the MIRIAM Registry [45] which powers Identifiers.org, thereby enabling users to select either the provider IRI (if available), the Identifiers.org IRI (if available), or the Bio2RDF IRI (for all data and ontologies) [46].

#### *Environmental ontologies for metagenomics*

In the domain of metagenomics, establishing a semantically controlled description of a sample’s original environment is essential for reliably archiving and retrieving relevant datasets. The BioHackathon resulted in a strategy for the re-engineering of the Metagenome Environment Ontology (MEO) [47], closely linked to the MicrobeDB project [48], to serve as community-specific portal to resources such as the Environment Ontology (EnvO) [49]. In this role, MEO will deliver curated, high-value subsets of such resources to the (meta)genomics community for use in efficient, semantically controlled annotation of sample environments. Additionally, MEO will enrich and shape the ontologies and vocabularies it references through persistently consolidating and submitting feedback from its users.

#### *An ontology for lexical resources*

The Life Science Dictionary (LSD) [50] consists of various lexical resources including English-Japanese/Japanese-English dictionaries with >230,000 terms, a thesaurus using the MeSH vocabulary [51,52], and co-occurring data that show how often a pair of terms appear in a MEDLINE [53] entry. LSD has been edited and maintained by the LSD project since 1993 and provides a search service on the Web, as well as a downloadable version. To assist with machine-readability of this important lexical resource, the group developed an ontology for this dataset [54], and an RDF serialization of the LSD was designed and coded at the BioHackathon. As a result, a total of 5,600,000 triples were generated and made available at the SPARQL endpoint [55].

#### *An ontology for incomplete enzyme reaction equations*

Incomplete enzyme reactions are not of interest to International Union of Biochemistry and Molecular Biology (IUBMB; who manage EC numbers) [56], but are common in metabolomics. Enzymes and reactions are described in Gene Ontology (GO) [57] and Enzyme Mechanism Ontology (EMO) [58], but they just follow the classification of IUBMB. It would be helpful to establish a structured representation to describe the available knowledge out of the reaction of interest even if the equation is not complete. Semantic representation of incomplete enzyme reaction equations was designed based on ontological principles. About 6,800 complete reaction equations taken from the KEGG [59,60] database were decomposed into 13,733 incomplete reactions, from which 2,748 chemical transformation patterns were obtained. They were classified into a semantic data structure, consisting of about 1,100 terms (functional groups, substructures, and reaction types) commonly used in organic chemistry and biochemistry. We keep curating the ontology for incomplete enzyme reaction equations aiming at its use in metabolome and other omics-level researches (available at GenomeNet [61]).

### Metadata

Metadata activities at the BioHackathon could be grouped into three areas of focus: service quality indicators, database content descriptors, and a broader inclusive discussion of generic metadata that could be used to characterize datasets in a database catalogue for enhanced data discovery, assessment, and access (not limited to but still useful for biodatabases).

#### *Service quality indicators*

With respect to data quality, the BioHackers coined the phrase “Yummy Data” as a shorthand way of expressing not only data quality, but more importantly, the ability to explicitly determine the quality of a given dataset. While quality of the published data is an important issue, it is a domain that depends as much on the underlying biological experiments as the code that analyses them. As such, the data quality working group at the BioHackathon focused on the issue of testing the quality of the published data endpoint, with respect to endpoint availability and other metrics. Therefore, the Yummy Data project [62] was initiated that periodically inspects the availability, response time, content amount and a few quality metrics for a selection of SPARQL endpoints of interest to biomedical investigators. While neither defining, nor executing, an exhaustive set of useful quality-measurements, it is hoped that this software may act as a starting point that encourages others to measure the “yumminess” of the data they provide, and thereby improve the quality of the published semantic resources for the global community.

#### *Database content descriptors*

The BioDBCore project [63,64] has created a community-defined, uniform, generic description of the core attributes of biological databases that will allow potential users of that database to determine its suitability for their task at hand (e.g. taxonomic range, update frequency, etc.). The proposed BioDBCore core descriptors are overseen by the International Society for Biocuration (ISB) [65], in collaboration with the BioSharing initiative [66]. One of the key activities of BioDBCore discussion at the BioHackathon was to define the RDF Schema and relevant annotation vocabularies and ontologies capable of representing the nature of biological data resources. As mentioned above, RDF representations necessitate the choice of a stable URI for each resource. The persistent identifiers considered for biological databases included NAR database collection [67,68], DBpedia [69,70], Identifiers.org and ORCID [71], while vocabularies from Biositemaps [72], EMBRACE Data and Methods (EDAM) [73], Biomedical Resource Ontology (BRO) [74] and The Ontology for Biomedical Investigations (OBI) [75] were evaluated to describe features such as resource and data types, and area-of-research. The exploration involved several specific use cases, including METI Life science integrated database portal (MEDALS) [76] and NBDC/DBCLS [77]. Another key activity at the hackathons was focused on the BioDBCore Web interface [78], both for submission and retrieval. Open issues include how to specify the useful interconnectivity between databases, for example, in planning cross-resource queries, and how to describe the content of biological resources in a machine-readable way to make it easily queried by SPARQL even if the vocabularies of any given resources are used. Currently, the group is considering the idea of using the named graph of a resource to store these kinds of metadata. There was also inter-group discussion of how to integrate BioDBCore with other projects such as DRCAT [79], which defines a similar, overlapping set of biological resources and their features.

#### *Generic metadata for dataset description*

The generic metadata discussion started by defining the problem of making database catalogue metadata machine-readable, so that a given dataset is automatically discoverable and accessible by machine agents using SPARQL. We discussed a set of conventions to describe the nature and availability of datasets on the emerging life science Semantic Web. In addition to basic descriptions, we focused our effort on elements of origin, licensing, (re-)distribution, update frequency, data formats and availability, language, vocabulary and content summaries. We expect that adherence to a small number of simple conventions will not only facilitate discovery of independently generated and published data, but also create the basis for the emergence of a data marketplace, a competitive environment to offer redundant access to ever higher quality data. These discussions have continued in teleconferences hosted by the W3C Health Care and Life Sciences Interest Group (HCLSIG) [80], and included at various times stakeholders such as DBCLS, MEDALS, BioDBCore, Biological Linked Open Data (BioLOD) [81], Biositemaps, UniProt, Bio2RDF, Biogateway [82], Open PHACTS [83], EURECA [84] and Identifiers.org.

### Platforms

#### *RDFization tools*

Generation of RDF data often requires iterative trials. In an early stage of prototyping RDF data, it is recommended to use OpenRefine [85] (formerly known as Google Refine) with the RDF extension [86] for correcting fluctuations of data, generation of URIs from ID literals and eventually converting tabular data into RDF. To automate the procedure, various hackathon initiatives generated RDFization tools and libraries, particularly for the Bio* projects. A generic tool, bio-table [87], can be used for converting tabular data into RDF, using powerful filters and overrides. This command-line tool is freely available as a biogem package and expanded during the BioHackathon to include support for named columns. Another Ruby biogem binary and library called bio-rdf [88] utilizes bio-table and generates RDF data from the results of genomic analysis including gene enrichment, QTL and other protocols implemented in the R/Bioconductor. The BioInterchange was conceived and designed during BioHackathon 2012 as a tool, web services and libraries for Ruby, Python and Java languages to create RDF triples from files in TSV, XML, GFF3, GVF and other formats including text mining results. User can specify external ontologies for the conversion and the project also developed biomedical ontologies of necessity for GFF3 and GVF data [89]. ONTO-PERL [90], a tool to handle ontologies represented in the OBO format, was extended to allow conversion of Gene Ontology (GO) annotations as RDF (GOA2RDF). Moreover, given that most legacy data resources have a corresponding XML schema, some effort was put into exploring and coding automated Schema-to-RDF translation tools for many of the widely used bioinformatics data formats such as BioXSD [91]. After working with the EDAM developers at the BioHackathon to modify their URI format to fit more naturally with an RDF representation, the EDAM ontology was successfully used to annotate the relevant portions of an automated BioXSD transformation, suggesting that significantly greater interoperability between bioinformatics resources should soon be enabled.

#### *Triple stores*

Moving from individual endpoints to multi-resource integration, the BioHackathon working group on triplestores also explored the problem of deploying multiple, interdependent and distributed triplestores, as well as searching over these, which included the examination of cluster-based triplestores, Hadoop-based triple stores [92-94], and emergent federated search systems. The group determined that Hadoop-based stores were not mature enough to be used for production use because it works with only limited types of data, and lacks functionality such as exposing a SPARQL endpoint, user interface, and so on. Regarding cluster-based triplestores, the group found that there was insufficient documentation regarding installation so this could not be tested sufficiently. Federated search using SPARQL 1.1 [95] could only be tested on OWLIM [96] at the time, and it was found that queries could not work efficiently across multiple endpoints. Thus, while single-source semantic publication seems to be well supported, the technologies backing distributed semantic datasets - both from the publisher’s and the consumer’s perspective - are lacking at this time.

### Applications

#### *Semantic Web exploration and visualization*

The Semantic Web simplifies the integration of heterogeneous information without the need for a pre-coordinated comprehensive schema. As a trade-off, querying Semantic Web resources poses particular challenges: how can a researcher understand what is in a knowledge base, and how can he or she understand its information structure enough to make effective queries? Interactive exploration and visualization tools offer intuitive approaches to information discovery and can help applied researchers to effectively make use of Semantic Web resources. In the previous edition of the BioHackathon, a working group focused on the development of prototypes to visualize RDF knowledge bases. As Semantic Web and Linked Data resources are becoming more available, in the life sciences and beyond, several new tools (interactive or not) for visualization of these kinds of resources have been proposed. The 2011 edition of the BioHackathon has created a review of such available tools, in view of their applicability in the biomedical domain. Through inspections and surveys we have gathered basic information on more than 30 tools currently available. In particular we have gathered information on:

##### 

**Requirements and availability** The operating systems supported, hardware requirements, licensing and costs. Relevant to an applied biomedical domain, we have also considered the availability of simplified install procedures.

##### 

**Features** The type of data access supported (e.g., via SPARQL endpoint or files-based), the type of query formulation supported (creating of graphic patterns, text based queries, boolean queries), whether some reasoning services is provided or exploited. Finally, when possible we have recorded some indication of type of user interaction proposed (e.g., browsing versus link discovery).

##### 

**Assistance and support** Whenever possible, we have collected information on the availability of community-based or commercial support, the availability of documentations, the frequency of software updates and the availability of user groups and mailing lists, for which we have sketched approximate activity metrics.

##### 

**Technical aspects** Whether the observed tools can be embedded in other systems, or if they provide a plugin architecture. When relevant, in which language they are developed, and finally which standards they support (e.g., VoID [97], SPARQL 1.1).

##### 

**Specificity to life sciences use cases** Finally, we have tried to collect information highlighting the usability of these tools in life sciences research (e.g., life sciences bundled datasets, relevant demo cases, citations per research area).

This collection of information is useful to decide which tools are potentially usable given constraints of technical, expertise or reliability nature. Following this data collection exercise, we have started to devise a classification of tools, by identifying some defining key characteristics. For instance, a key characteristic of the surveyed tools is their approach to data: some focus more on instance data and tend to provide a graph-like metaphor. Some focus more on classes and relations and tend to present a class-based access. Another key aspect is the degree to which visualization tools aim at supporting data exploration, rather than explanation. Based on our classification, we aim at choosing a few representative tools, provide some benchmarking and evaluate how different types of tools are effective in simple tasks.

#### **Ontology mapping visualization**

Ontology mapping deals with relating concepts from different ontologies and is typically concerned with the representation and storage of mappings between the concepts [98]. BioPortal ontologies [40] are usually interconnected, and mappings between them are available, although a visualization of these mappings is not currently available. Two types of mapping visualizations were explored at the BioHackathon: (1) A visualization of ontology mappings of all BioPortal ontologies, and (2) A visualization of a subset of BioPortal ontologies that would be useful in OntoFinder/Factory [99] - a tool for finding relevant BioPortal ontologies and also building new ontologies. The hackers investigated the applicability and utility of two tools/environments: Google Fusion Tables [100], and Gephi [101]. This work is ongoing.

#### **Identifier conversion service**

The existence of multiple synonyms for the same data (sets) often inhibits cross-resource querying and data mining. Thus, a centralized server containing curated links between and among life-science databases would greatly facilitate the data integration tasks in bioinformatics. The members of the G-language [102] group began developing an identifier conversion Web service named G-Links. Based on the cross referencing information available from UniProt and KEGG, this RESTful service retrieves all identifiers and their corresponding PURLs related to an identifier provided by the user. In addition, users may supply nucleotide or amino acid sequences in place of the identifier, for rapid annotation of sequences. In order to comply with the recent Semantic Web and Linked Data initiatives, results can be returned in N-triples or RDF/XML formats for interoperability, as well as the legacy GenBank, EMBL and tabular formats (Table 2). This service is freely available at http://link.g-language.org/.

**Table 2:** Example queries using G-Links

**Query**	**REST API**
GeneID:947170 by tabular format	http://link.g-language.org/GeneID:947170
P0A7G6 (UniProt) by N-Triple format	http://link.g-language.org/P0A7G6/format=nt
hsa:126 (KEGG) by RDF format	http://link.g-language.org/hsa:126/format=rdf
POST sequence directly	https://gist.github.com/1172846

One of the central advantages of Linked Data as an end-user biologist is the ease of discovery and retrieval of related information. On the other hand, biological data is highly inter-related, and the multitude of linkages can easily become overwhelming, resulting in familiar “hair balls” frequently seen in protein-interaction networks. Sophisticated filtering of Linked Data result sets, ranking the results according to relevance to one’s interests, or by some form of enrichment of interesting phenomena would assist greatly in interpreting the content of semantic data stores. Such filtering, or data arrangement and presentation, should ideally be accompanied by an intuitive visualization. Participants pursued these goals by first generating a complete genome (gene set) of *Escherichia coli* as Linked Data using G-Links, together with several associated numerical datasets calculated through the G-language REST Web service [103] (a product of BioHackathon 2009). Statistics such as Cramer’s V for nominal data and Spearman’s rank correlation for continuous data were applied to data coming from multiple, overlapping sources (e.g. KEGG versus Reactome [104] versus BioCyc [105] for pathways) to cluster result sets according to their similarity. This would allow, for example, a user to choose the least-redundant subset of results in order to maximize the amount of unique information passed to a visualization tool. Using the inverse, these metrics can be used to screen for enrichment, where over-representation of the same dataset is considered meaningful, and therefore that dataset should be highlighted. An example of both types of filtering was created by the participants using the JavaScript InfoViz Tookit [106]. The resulting graph is highly interactive, and all nodes representing data sets can be clicked to re-layout the graphs centering to the clicked data set, with animations. Demonstrations using pre-calculated *E. coli* data are available [107,108].

#### *Natural language semantic query via voice recognition*

Finally, the project that generated the most “buzz” among the participants in BioHackathon 2012 was Genie - a “Siri [109] for Biologists”. The G-language Project members undertook the development of a virtual research assistant for bioinformatics, designed to be an intuitive entry-level gateway for database searches. The prototype developed and demonstrated at the BioHackathon was limited to gene- and genome-centric questions. Users communicate with Genie using spoken English, and Genie replies in a synthesized voice. Genie can find information on three main categories: 1. Anything about a gene of interest, such as, what is the sequence, function, cellular localization, pathway, related disease, related SNPs and polymorphisms, interactions, regulations, expression levels; 2. Anything about a set of genes, based on multiple criteria. For example, all SNPs in genes that are related to cancer, that work as transferases, that are expressed in the cytoplasm, and that have orthologs in mice; 3. Anything about a genome, such as, production of different types of visual maps, calculation of GC skews, prediction of origins and terminus of replication, calculation of codon usage bias, and so on. Using an NLP and dictionary-based approach, with the species name as a top-level filter to reduce the search/retrieval space, annotations are fetched for this species, and a dictionary of gene names is created dynamically. In order to implement integrated information retrieval, the following software systems were used:

• The G-language Genome Analysis Environment and its REST service which allows for extremely rapid genome-centric information retrieval.

• G-language Maps (Genome Projector and Pathway Projector, as well as Chaos Game Representation REST Service) which visualizes that genomic information.

• Keio Bioinformatics Web Services EMBASSY package and EMBOSS [110], which provides more than 400 tools that can be applied to the information.

• G-Links - an extremely rapid gene-centric data aggregator.

The Genie prototype is accessible online [111,112].

## Conclusions

BioHackathon series started out with the Integrated Database Project of Japan, aiming to integrate all life science databases in Japan. Initially, the focus was on Web services and workflows to enable efficient data retrieval. However, the focus eventually shifted towards Semantic Web technologies due to the increasing heterogeneity and interlinked nature of the data at hand, for example, from the accumulation of next-generation sequencing data and their annotations. From this, the community recognized the importance of RDF and ontology development - fundamental Semantic Web technologies that have also come to gain the attention of other domains in the life sciences, including genome science, glycosciences and protein science. For example, BioMart and InterMine, which were initially developed to aid the integration of life science data, has now started to support Semantic Web technologies. These hackathons have served as a driving force towards integration of data “islands” that have slowly started linking to one another through RDF development. However, insufficient guidelines, ontologies and tools to support RDF development has hampered true integration. The development of such guidelines, ontologies and tools has been the central focus of these hackathons, bringing together the community on a consistent basis, and we have finally started to grow buds from these efforts. We expect to bear fruit in the near future by the development of biomedical and metagenome applications on top of these developments. Moreover, we expect that text mining will become increasingly vital to enriching life science Semantic Web data with the knowledge currently hidden within the literature.

## Abbreviations

ASCII: American Standard Code for Information Interchange; BCSDB: Bacterial Carbohydrate Structure Database; BRO: Biomedical Resource Ontology; CAI: Codon Adaptation Index; DBCLS: Database Center for Life Sciences; DOI: Digital Object Identifier; DRCAT: Data Resource CATalogue; EDAM: EMBRACE Data And Methods; EMBOSS: European Molecular Biology Open Software Suite; EnvO: Environment Ontology; EURECA: Enabling information re-Use by linking clinical REsearch and Care; FALDO: Feature Annotation Location Description Ontology; FOP: Frequency of OPtimal codons; GAE: Genome Analysis Environment; GFF3: Generic Feature Format version 3; GFF2OWL: Generic Feature Format to Web Ontology Language; GO: Gene Ontology; GOA2RDF: Gene Ontology Annotations to RDF; INSDC: International Nucleotide Sequence Database Collaboration; IRI: Internationalized Resource Identifier; ISB: International Society for Biocuration; IUBMB: International Union of Biochemistry and Molecular Biology; JCGGDB: Japan Consortium for Glycobiology and Glycotechnology Database; KEGG: Kyoto Encyclopedia of Genes and Genomes; LSD: Life Science Dictionary; MEDALS: METI DAtabase portal for Life Science; MEO: Metagenome Environment Ontology; MeSH: Medical Subject Headings; MIRIAM: Minimal Information Required In the Annotation of Models; NBDC: National Bioscience Database Center; NLP: Natural Language Processing; NCBO: National Center for Biomedical Ontology; OBI: Ontology for Biomedical Investigations; OBO: Open Biomedical Ontology; Open PHACTS: Open Pharmaceutical Triple Store; OWL: Web Ontology Language; PDF: Portable Document Format; PHX: Predicted Highly eXpressed genes; PURL: Permanent URL; RDF: Resource Description Framework; REST: REpresentational State Transfer; RINGS: Resource for INformatics of Glycomes at Soka; SIO: Semanticscience Integrated Ontology; SNPs: Single Nucleotide Polymophisms; SO: Sequence Ontology; SPARQL: SPARQL Protocol and RDF Query Language; UUID: Universally Unique Identifier; XML: eXtensible Markup Language.

## Competing interests

The authors declare they have no competing interests.

## Authors’ contributions

TK, MDW, and KFA primarily wrote the manuscript based on the group summaries written by participants. TK, SK, YY, AY, SO, SK2, JK, YW, HW, YK, HO, HB, SK3, SK4, TT organized BioHackathon 2011 and/or 2012. All authors except for JA and NP attended the BioHackathon 2011 and/or 2012. All authors read and approved the final manuscript.
